# Length-independent charge transport of well-separated single-crystal TiO_2_ long nanowire arrays†
†Electronic supplementary information (ESI) available. See DOI: 10.1039/c8sc02335b


**DOI:** 10.1039/c8sc02335b

**Published:** 2018-08-06

**Authors:** Jie Liu, Xia Sheng, Fengying Guan, Ke Li, Dandan Wang, Liping Chen, Xinjian Feng

**Affiliations:** a College of Chemistry , Chemical Engineering and Materials Science , Soochow University , Suzhou 215123 , China . Email: xjfeng@suda.edu.cn

## Abstract

Long, well-separated single crystal TiO_2_ nanowire (NW) arrays with rapid charge transport properties hold great promise in photoelectrochemical and energy storage devices. Synthesis variations to increase the NWs length generally result in the widening of the NWs and fusion at their roots which, in turn, increases the structural disorder and slows charge transport. As such, well-separated single-crystal TiO_2_ NW arrays with rapid charge transport properties have been limited to lengths of about 3–4 μm. In this work, by adjusting the HCl/DI-water ratio and adding specific organic ligands to the reaction solution that slow the lateral growth rate we achieve well-separated single-crystal rutile TiO_2_ NW arrays with a length of ∼10 μm and an aspect ratio of approximately 100. The charge transport is 100 times faster than that of nanoparticle films and remarkably exhibits length-independence, a behavior that can be attributed to the well-separated architecture. The synthesis strategy can be extended to the fabrication of other well-separated metal oxide NW arrays and represents an important tool in achieving high performance photoelectrochemical and electrical energy storage devices.

## Abstract 2

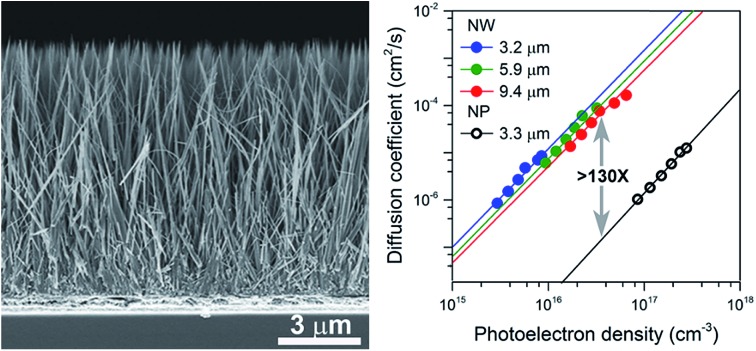
Vertically aligned and well-separated single-crystal TiO_2_ NW arrays with a length of up to ∼10 μm were synthesized *via* a simple solvothermal method. The charge transport in these NWs is over 100 times faster than that of nanoparticle films and remarkably exhibits length-independence.

## Introduction

Vertically aligned semiconductor nanowire (NW) arrays have attracted great attention due to their unique optical and electronic properties.^1^–^8^ Recent work on well-separated single-crystal rutile TiO_2_ NW arrays has shown that the electron diffusion coefficient is two orders of magnitude higher than that in mesoporous films comprised of randomly packed nanoparticles (NPs), corresponding to an electron diffusion length of about 60 μm^9^ and offering the promise of useful application of long NW arrays within devices such as solar cells, photodetectors, and chemical sensors.^10^–^13^ However, the synthesis of well-separated rutile TiO_2_ NW arrays has been limited to lengths of about 3–4 μm.^7^,^14^,^15^ During the conventional bottom-up growth process, synthesis variations to increase the length generally result in the widening of the NWs and subsequent fusion at their roots.^16^–^18^ This, in turn, reduces the aspect ratio, increases the structural disorder and reduces the charge transport;^19^,^20^ experimental results shown in ESI Fig. S1† indicate that the diffusion coefficient of bundled TiO_2_ NWs is over one order of magnitude lower than that of well-separated ones. To date, the growth of long and well-separated single-crystal rutile TiO_2_ NW arrays has remained an unmet challenge.

Herein, by suppressing NW lateral growth, we report the one-step synthesis of well-separated single-crystal rutile TiO_2_ NW arrays with a length of ∼10 μm having an aspect ratio of approximately 100, a value over 5 times greater than previously reported.^7^ Not only is electron transport in these NW arrays 100 times faster than in NP films, but charge collection efficiency is found to be independent of length. We believe that the well separated long single-crystal TiO_2_ NW arrays can facilitate the development of a broad range of photovoltaic and photoelectrochemical devices.

## Results and discussion

### Characterization of TiO_2_ NW arrays

**Fig. 1:**
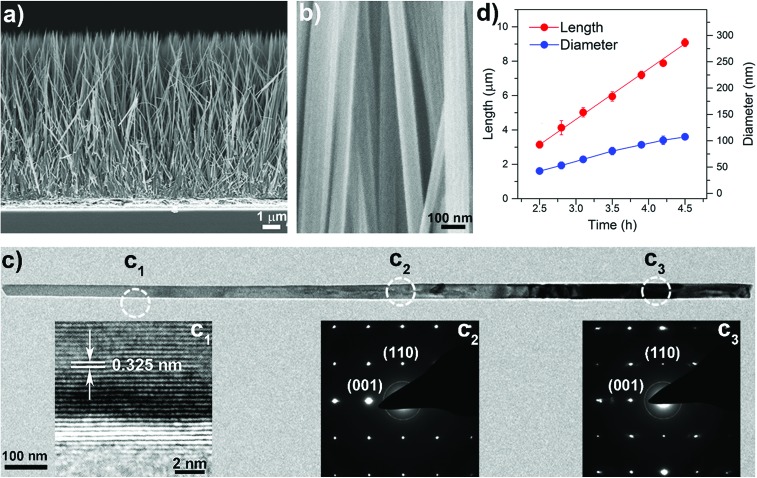
Microstructure characterization of the long and well-separated TiO_2_ NWs. (a) and (b) are FE-SEM cross-sectional images of the NWs grown on a FTO-coated glass substrate at low and high magnifications, respectively. (c) TEM images of part of a single NW. Insets in panel (c) are, respectively, the HR-TEM image (c_1_) and SAED patterns (c_2_ and c_3_) recorded from their corresponding regions. The same crystallographic orientation at different areas indicates that the NW is a single crystal. (d) Length and diameter of the TiO_2_ NWs *versus* growth time. Lines are fits to the data, and error bars represent one standard deviation.

Aligned well-separated ∼10 μm long single-crystal rutile TiO_2_ NW arrays were prepared *via* a facile solvothermal method. In a typical experiment, a FTO coated glass substrate supporting a TiO_2_ seed layer was loaded into a Teflon-lined stainless-steel reactor (23 mL) filled with a reaction solution composed of 2-butanone, ethanol, hydrochloric acid (HCl, 37%) and titanium butoxide (TBOT). The reaction temperature was set at 443 K for variable durations. Fig. 1a and b are typical field emission scanning electron microscope (FE-SEM) images of the NWs obtained after a reaction time of 4.5 h. The NWs have an average length of ∼10 μm, 98–105 nm diameters, and an aspect ratio of ∼100, and are well separated over their entire length. From X-ray diffraction (XRD), see Fig. S2,† the crystal phase of the NWs can be identified as tetragonal rutile (JCPDS no. 21-1276). The (002) diffraction peak is much stronger than the other peaks, suggesting that the NWs have a preferred [001] growth direction. Fig. 1c is a transmission electron microscopy (TEM) image of a typical NW. The high-resolution TEM image shown in the inset of Fig. 1c indicates that the NW is a single crystal with lattice fringes of 0.325 nm, which can be assigned to the inter-planar distance of rutile. The similar spot-features of the selected area electron diffraction (SAED) patterns, shown in the inset of Fig. 1c, along the NW length confirm a uniform single crystal structure. Fig. 1d shows the length and diameter of the NWs for different growth times. As the reaction time increases from 2.5 to 4.5 h the length linearly increases from about 3 to 10 μm (see Fig. S3†), while the NW diameters remain within a narrow range of 50–105 nm.

### Growth mechanism

**Fig. 2:**
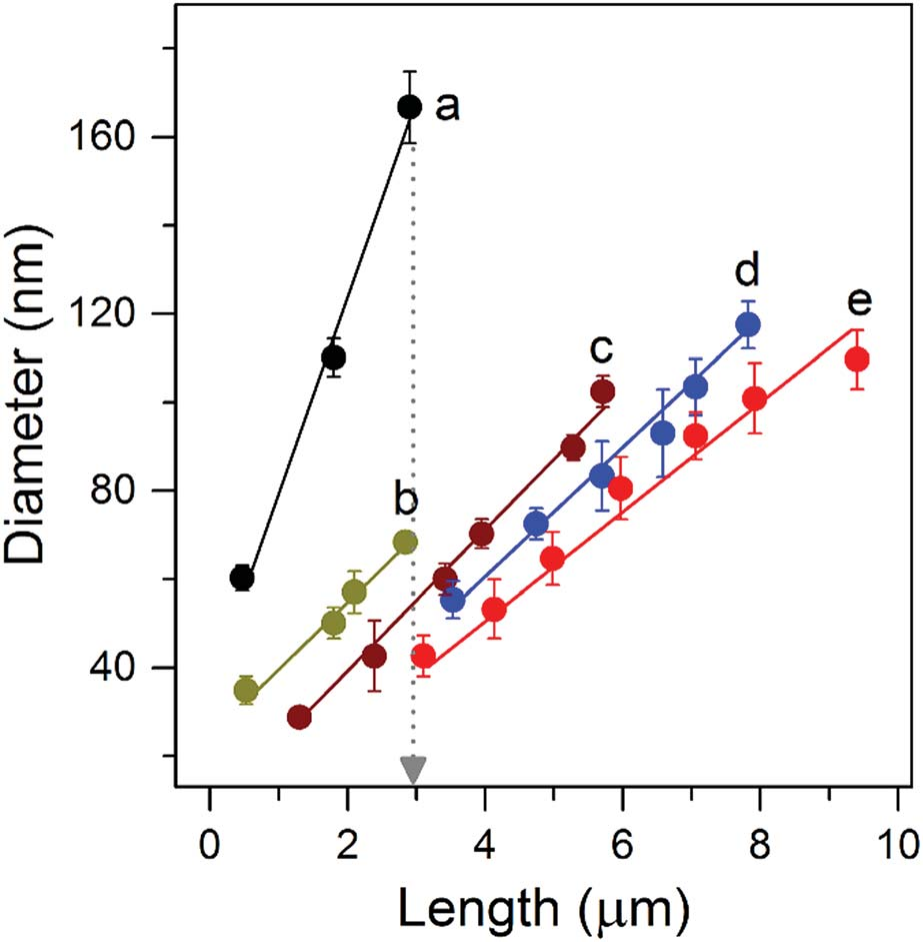
Diameter *versus* length of NWs grown using the indicated reaction solutions (see Table 1). Lines are fits to the data, and error bars represent one standard deviation.

The suppression of lateral growth is the key to synthesizing long and well-separated NWs. To help understand the growth process experiments using various reactants (Table 1) were carried out. As shown in Fig. 2 (line a), Fig. S4a and S5a,† when a reaction solution composed of HCl (37%) and deionized (DI) water with a volume ratio of 1 : 1 was employed, isolated NWs of ∼3 μm length and ∼170 nm diameter were obtained, with an aspect ratio of ∼18, similar to results reported previously.^7^,^13^,^21^ During the hydrothermal process, Ti^4+^ will first hydrolyze and form anionic complexes of [Ti(OH)_*x*_Cl_*y*_]^2–^, where *x* + *y* = 6.^22^ TiO_2_ is then formed *via* a dehydration reaction between the OH ligands of these complexes. Meanwhile, selective absorption of Cl^–^ ions on the side facets of the NWs helps suppress linking between OH ligands, minimizing growth in the lateral direction.^23^ This suggests that a bigger *y* or a smaller *x* number, *i.e.*, a higher *y*/*x* value of the hydrolyzed complex is required to suppress the lateral growth and fusion of NWs, thereby obtaining long and well-separated NW arrays of a high aspect ratio.

**Table 1:** Reaction solution compositions, see Fig. 2

No.	*a*	*b*	*c*	*d*	*e*
HCl (mL)	6	10	10	10	10
H_2_O (mL)	6	2	1	0	0
2-Butuanone (mL)	0	0	1	2	1.9
Ethanol (mL)	0	0	0	0	0.1
Best aspect ratio	18.8	48.8	63.4	78.2	94.1

To increase the *y*/*x* value and suppress the lateral growth we first increased the volume ratio of HCl/DI water from 1 : 1 to 5 : 1, which increases both Cl^–^ and H^+^ concentrations and thus has two main effects on the growth process. (1) Increasing the Cl^–^ concentration can suppress the lateral growth as it can selectively be absorbed on the side facets; (2) increasing the H^+^ concentration can lower *x* in the [Ti(OH)_*x*_Cl_*y*_]^2–^complex, giving a bigger value of *y* and the *y*/*x* ratio. As such, the lateral growth of the NWs is significantly suppressed. The striking effect of this strategy is seen by plotting NW length *versus* diameter for different growth times (Fig. 2, line b). Isolated NWs with a much smaller diameter of ∼70 nm and an aspect ratio of ≈ 48 are obtained (Fig. S4b and S5b†). To further increase the *y*/*x* value polar organic solvents were used instead of DI water. In the presence of organic solvents, such as 2-butanone, some of the OH ligands in the complex will be substituted, in turn reducing *x* and thereby increasing the *y*/*x* ratio and suppressing the dehydration reaction in the lateral direction. As shown in Fig. 2 (lines c and d), S4 (c and d) and S5 (c and d),† the NW diameter decreased when DI water in the reaction solution was replaced with 2-butanone allowing the extended growth of high aspect ratio NWs. Subsequently, ethanol was used to replace part of the 2-butanone in order to replace the OH ligands in the complex, reduce the *x* number and thereby increase the *y*/*x* ratio. As expected, due to the smaller molecule size of ethanol, well-separated NWs with a much smaller diameter and a length up to ∼10 μm were obtained, see Fig. 2 (line e), S4e and 1d. The highest aspect ratio is ∼100, a value five times greater than that achieved using conventional reaction solutions.^7^ Our results reveal that the effective suppression of lateral NW growth during the solvothermal growth process is the key factor for obtaining long and well-separated NW arrays.

### The photoelectrochemical properties of the TiO_2_ NW arrays

**Fig. 3:**
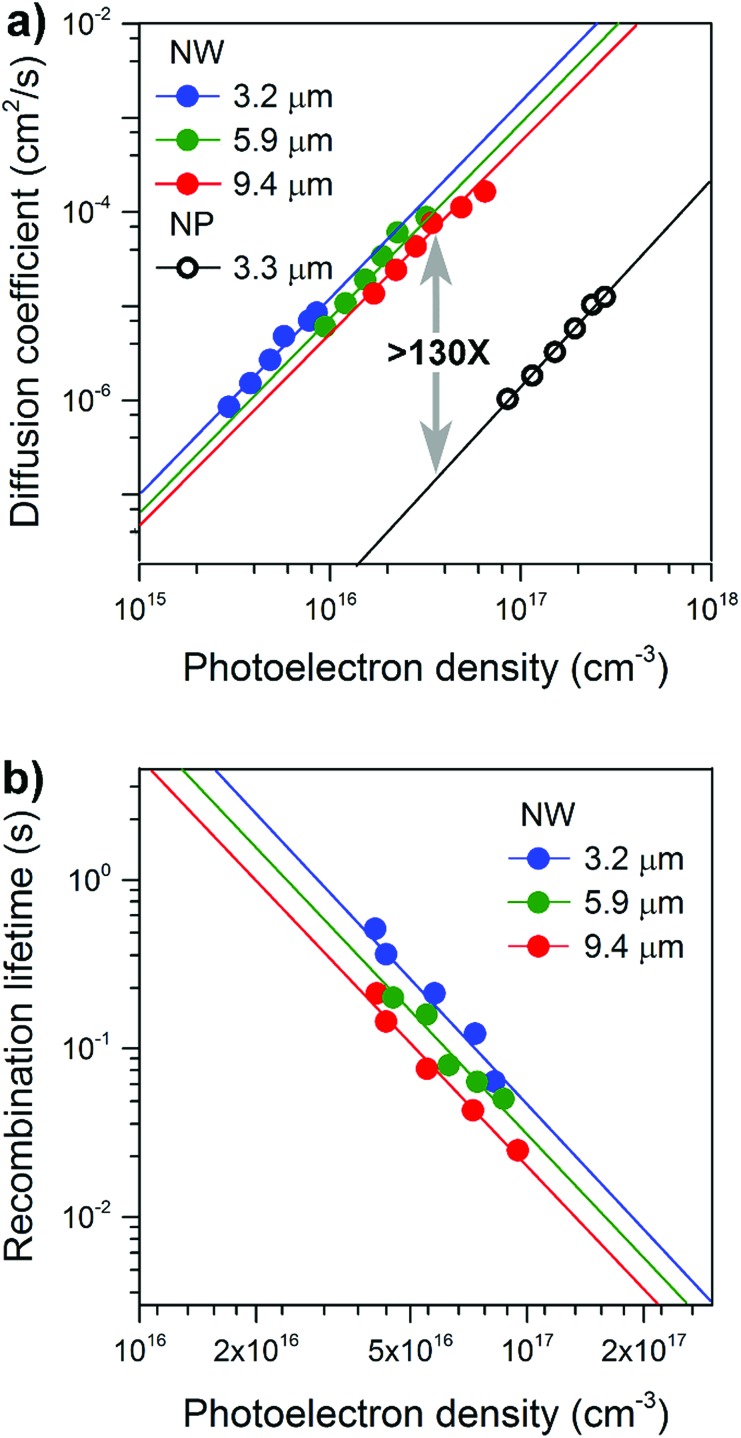
Electron transport and recombination dynamics of NW-based photoelectrodes of different thicknesses. (a) Comparison of electron diffusion coefficients as a function of the photoelectron density for NW and NP based photoelectrodes. (b) Comparison of recombination lifetimes as a function of photoelectron density for NW based photoelectrodes.

The photoelectric properties of the well-separated single-crystal TiO_2_ NWs were explored using intensity-modulated photocurrent spectroscopy (IMPS) and intensity-modulated photovoltage spectroscopy (IMVS) techniques.^24^,^25^ Fig. 3a compares the photoelectron density (*n*) dependence of the electron diffusion coefficient (*D*) for NW and NP films. The morphology of the NP films is shown in Fig. S6.† The values of *D* and *n* were determined using procedures described in detail elsewhere.^26^,^27^

The electrode *D* values display a power-law-dependence with *n*, which can be attributed to the electrons undergoing multiple trapping-detrapping events within an exponential distribution of conduction band tail states.^28^ Over a broad range of charge densities, as the NW length increased from 3.2 to 9.4 μm no obvious decrease in *D* was observed; *D* values are all over 100-times higher than that of the NP films. Fig. 3b compares the photoelectron density (*n*) dependence of the recombination time (*τ*_r_) for NW array-based solar cells. Comparable electron lifetimes are recorded for NWs of different lengths and the same photoelectron density of 10^17^ cm^–3^, suggesting that the number of surface trap states is almost the same. Our results reveal excellent charge transport capabilities that are length-independent, properties we attribute to the well-separated, single-crystal nature of the NWs and long-range structural coherence.

**Fig. 4:**
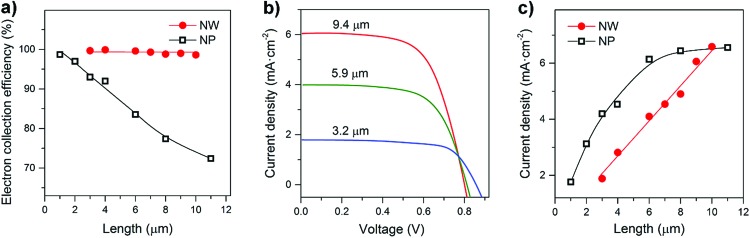
Performance of the rutile TiO_2_ NW and NP-based solar cells. (a) Dependence of the electron collection efficiency on the NW length and NP-film thickness. (b) Comparison of the current density–voltage characteristics of the NW-based cells under AM 1.5 illumination. (c) Dependence of current density on the NW length and NP-film thickness.

On the basis of the transport and recombination time constants shown in Fig. 3 and S7,† the charge collection efficiency (*η*_cc_) of NW arrays and NP films was calculated by using *η*_cc_ = 1 – *τ*_c_/*τ*_r_. As shown in Fig. 4a, the *η*_cc_ values of the NW arrays are all about 100%, exhibiting performance independent of film thickness (*d*). In contrast for the NP films there is an obvious decrease in *η*_cc_ as the film thickness increases. The independence of *d* and *η*_cc_ of NW arrays is of great importance to the performance of optoelectronic devices.

Fig. 4b and S8† compare the *J*–*V* characteristics of NW and NP-based dye sensitized cells under simulated AM 1.5 light. The short circuit photocurrent density (*J*_sc_) of the NW-based cells increases steadily with the NW length. In contrast, no obvious improvement in the NP-based cells is observed for film thicknesses greater than 6 μm. Fig. 4c presents the calibration plot of *J*_sc_*versus d*. In general, *J*_sc_ is determined by light-harvesting efficiency (*η*_lh_), charge-injection efficiency (*η*_inj_), and electron collection efficiency (*η*_cc_). The *J*_sc_ was estimated using *J*_sc_ = *qη*_lh_*η*_inj_*η*_cc_*I*_0_, where *q* is the elementary charge, and *I*_0_ is the incident photon flux. The *η*_lh_ value can be considered to be primarily dependent on the photoelectrode surface area,^29^ which was measured *via* dye desorption (see Fig. S9 and S10 and Tables S1 and S2†). From Fig. S11,† we can see that the surface areas of both NW and NP films increase linearly with the film thickness; since the *η*_cc_ of the NW films is independent of thickness, the *J*_sc_ value increases linearly with increasing NW length. In contrast, because the *η*_cc_ value of the NP-based cells decreases with *d*, their *J*_sc_ value tends to saturate as the film thickness increases. These results suggest that photoelectrodes possessing rapid charge transport properties and a charge collection efficiency independent of film thickness are of great importance to the development of high performance optoelectronic devices.

## Conclusions

We have developed a simple solvothermal method for the growth of vertically aligned and well-separated single-crystal TiO_2_ NW arrays with a length of up to 10 μm on FTO-coated glass substrates. The effective suppression of the lateral growth is key to the NW growth process, resulting in a length to diameter ratio of approximately 100. For the first time we show a material system for which the electron transport is independent of length, leading to a 100% collection efficiency even for 10 μm thick films. This result is of great importance for the practical application of photocatalytic and electrical energy storage systems. In particular, it is an ideal basis for photoelectrode design, and application, including metal nanoparticle (QD)/NW hybrids for plasmonic water splitting, CO_2_ photoreduction,^30^ and the construction of multi-bandgap (rainbow) photovoltaics.

## Experimental

### Preparation of TiO_2_ NW arrays

In a typical synthesis, the FTO coated glass substrates (Tech 7) were cleaned using DI water, acetone and ethanol. After being air dried, a TiO_2_ seed layer was deposited by dip-coating (0.4 M tetrabutyl titanate in ethanol) followed by 30 min heat treatment at 823 K in air. The TiO_2_-seeded FTO coated glass substrates were then loaded into Teflon-lined stainless-steel reactors (23 mL) filled with a solution mixture containing 1.9 mL 2-butanone, 0.1 mL ethanol, 10 mL 37% hydrochloric acid (HCl) and 1.3 mL titanium butoxide (TBOT) and kept at 443 K for a certain time. The treatments of the NW arrays were as follows: the as-grown TiO_2_ NW arrays were immersed in a fresh H_2_O_2_ (30 wt%)/NH_3_·H_2_O (25 wt%; v : v = 10 : 1) solution for 10 min and then rinsed with a copious amount of distilled water. After being dried in air, the NW arrays were annealed at 723 K for 30 min in an oxygen-rich environment with an oxygen flow rate of 0.6 L min^–1^ before all measurements. Finally, the rutile TiO_2_ NW arrays were allowed to cool to room temperature.

## Conflicts of interest

There are no conflicts to declare.

## Supplementary Material

Supplementary information
