# Supplementary material for: Length-independent charge transport of well-separated single-crystal TiO2 long nanowire arrays
Source: Chem Sci. 2018 Aug 6;9(37):7400–4. doi: 10.1039/c8sc02335b (PMC6237121; doi:10.1039/c8sc02335b)
Supplement: Supplementary file 1 [file SC-009-C8SC02335B-s001.pdf]

Electronic Supplementary Material (ESI) for Chemical Science.
This journal is © The Royal Society of Chemistry 2018

Supporting Information

Length-Independent Charge Transport of Well-Separated Single-Crystal TiO_2 Nanowire Arrays

Jie Liu, Xia Sheng, Fengying Guan, Ke Li, Dandan Wang, Liping Chen, Xinjian Feng*

College of Chemistry, Soochow University, Suzhou 215123, P. R. China

Materials and methods

Synthesis of rutile TiO_2 nanoparticle (NP) films. In a typical synthesis, titanium tetrachloride ($TiCl_4$) was used as starting material. It was added slowly into ice water under stirring to prepare a $TiCl_4$ (4.5 M) aqueous solution. The pH (pH=1) of the medium was adjusted using KOH. Then 13 mL of the $TiCl_4$ (1.0 M) solution was placed to a 23 mL Teflon-lined stainless-steel autoclave apparatus and kept at 493 K for 2 h. After the autoclave was cooled to room temperature, the suspension was centrifuged, washed with ethanol and dried at 353 K. The resulting NPs were then coated onto TiO_2-seeded FTO substrates followed by a 30 min heat treatment at 723 K.[1]

Assembly of dye sensitized solar cells (DSSCS). As-prepared samples were coated with dye by immersion for 24 h at ambient temperature in a 0.5 mM ethanolic solution of commercial N719 dye. The electrolyte is composed of 1,2-dimethyl-3-propylimidazolium iodide, lithium iodide, iodine, tributyl phosphate, guanidinium isothiocyanate. The counter electrode was fabricated by deposition of a thin layer of platinum. The thickness of the electrolyte layer between the rutile TiO_2 NWs and counter electrode was fixed by the use of a 25 μm thick Surlyn 1702 film spacer.

Characterization: The morphology of the TiO_2 NWs and assembled DSSCs were examined by SEM (S4700, Hitachi, Tokyo, Japan) and TEM (Tecnai G2 F20 S-Twin, FEI, USA, 200 kV). X-ray diffraction (XRD) analysis was performed using an X-ray powder diffractometer (X'Pert Pro MPD, Holland Panalytical). The photocurrent density and photovoltage of the DSSCs were measured with active sample areas of about 0.36 cm2 using a Xe lamp with AM-1.5 simulated sunlight filter. Electron transport and recombination properties were measured by intensity-modulated photocurrent spectroscopy (IMPS) and photovoltage spectroscopies (IMVS) on a photoelectrochemical measurement system (Modulab XM photoEchem, Solartron, UK).

References

1 H. Cheng, J. Ma, Z. Zhao and L. Qi, *Chem. Mater.,* 1995, ***7***, 663-671.

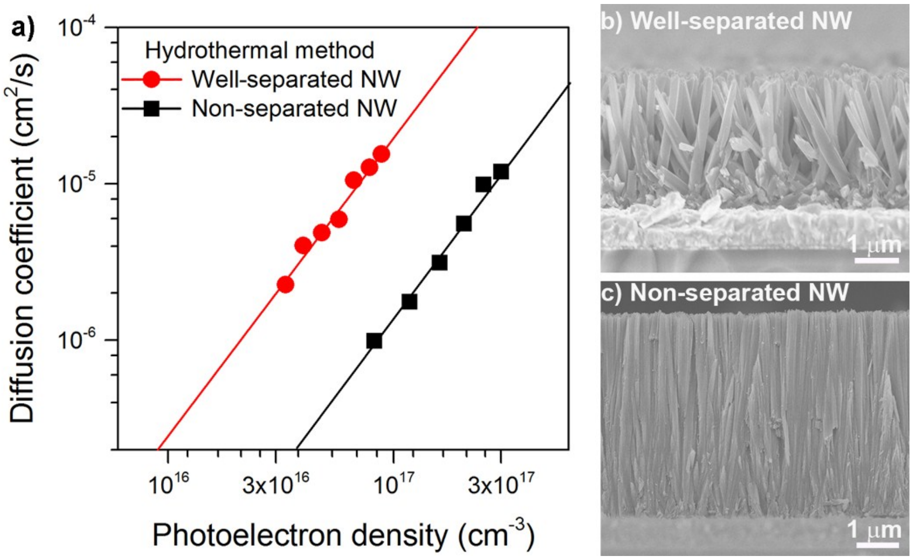

Figure S1. (a) Comparison of electron diffusion coefficients as a function of the photoelectron density for well-separated and non-separated TiO_2 NWs based cells. (b) and (c) are, respectively, SEM images of the well-separated and non-separated TiO_2 NWs. The well-separated NWs were prepared by using mixed reaction solutions of 6 mL HCl, 6 mL DI water and 0.2 mL TBOT at 453 K for 3h. The non-separated NWs were prepared by using mixed solutions of 6 mL HCl, 6 mL DI water and 0.4 mL TBOT at 453 K for 3h.

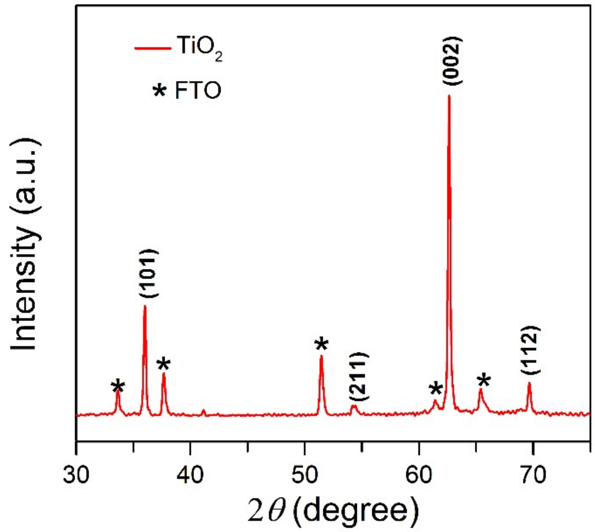

Figure S2. XRD pattern of the as-prepared NWs with a preferred [001] orientation.

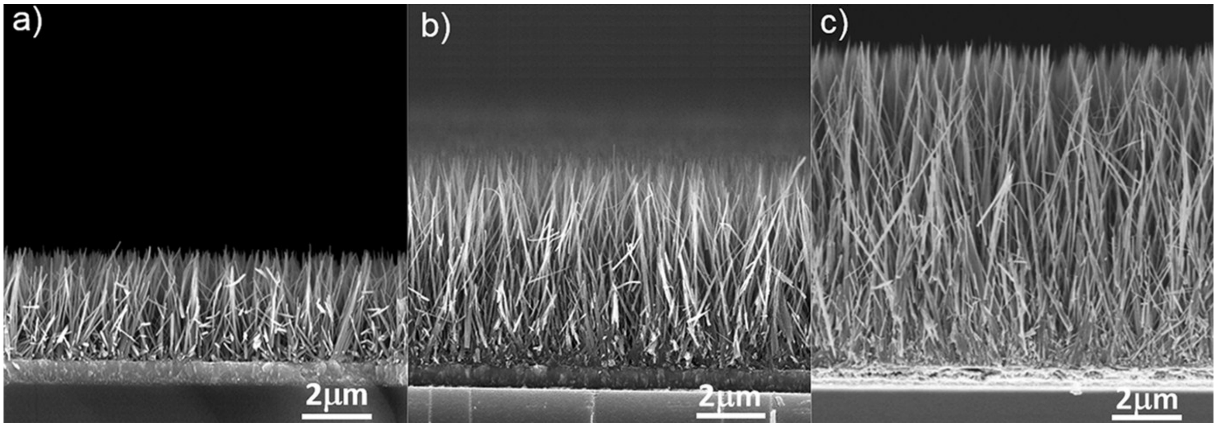

Figure S3. (a-c) SEM images of the TiO_2 NWs obtained after reaction times of 2.5 h, 3.5 h and 4.5 h.

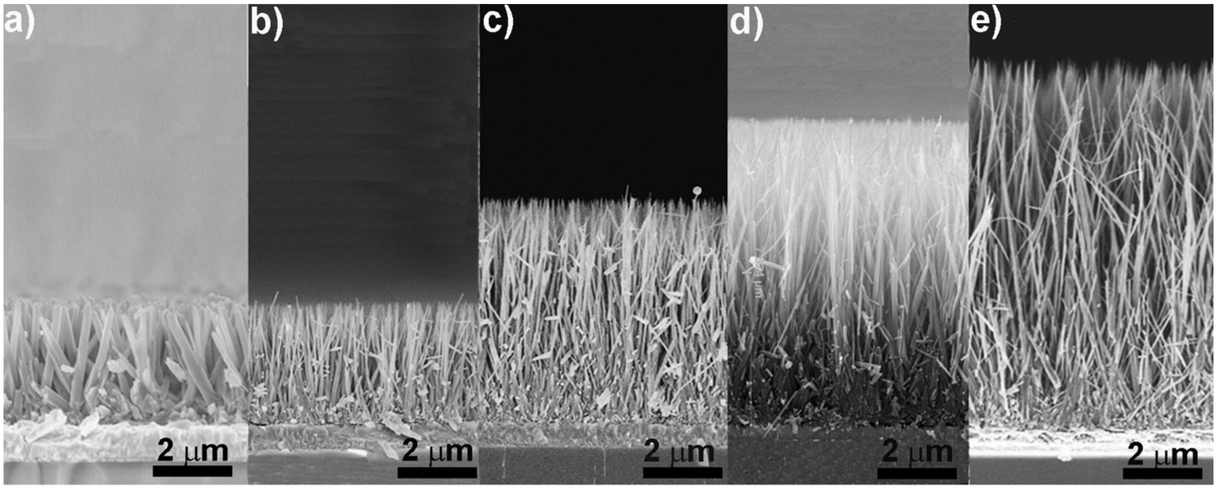

Figure S4. SEM images of TiO_2 NWs obtained under different growth conditions: (a) Synthesized by using a mixed solution of 6 mL HCl, 6 mL DI water and 0.2 mL TBOT at 453 K for a reaction time of 3 h. (b) Synthesized by using a mixed solution of 10 mL HCl, 2 mL DI water and 1.3 mL TBOT at 443 K for a reaction time of 2 h. (c) Synthesized by using a mixed solution of 10 mL HCl, 1 mL butanone, 1 mL DI water and 1.3 mL TBOT at 443 K for a reaction time of 3.5 h. (d) Synthesized by using a mixed solution of 10 mL HCl, 2 mL butanone, and 1.3 mL TBOT at 443 K for a reaction time of 4 h and 15 min. (e) Synthesized by using a mixed solution of 10 mL HCl, 1.9 mL 2-butanone, 0.1 mL ethanol, and 1.3 mL TBOT at 443 K for a reaction time of 4.5 h.

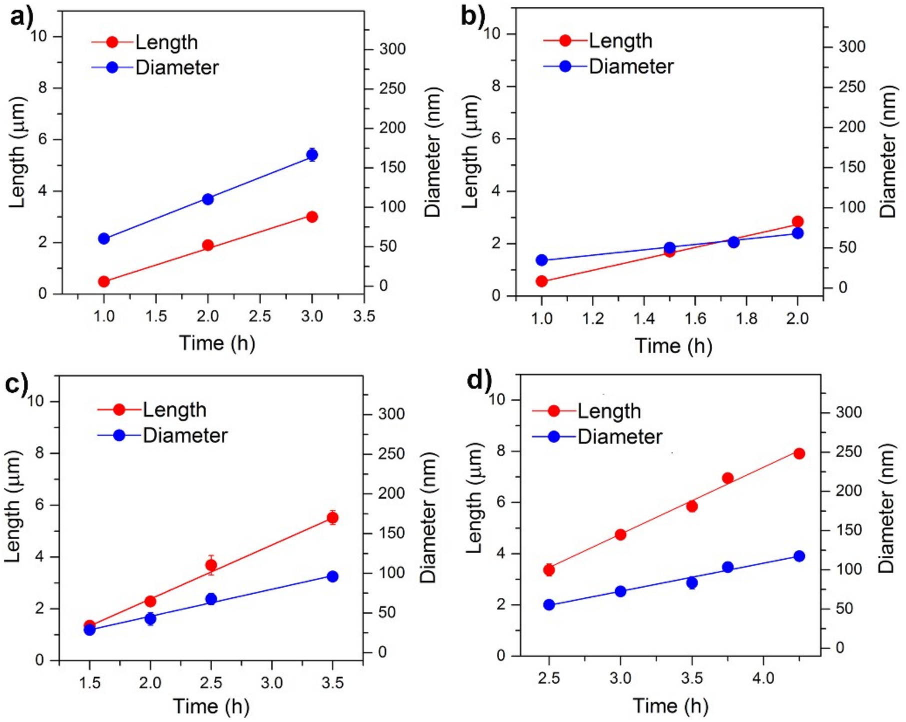

Figure S5. (a-d) Length and diameter of the TiO_2 NWs versus growth time under different growth conditions as shown in Table 1. Lines are fits to the data, and error bars represent one standard deviation.

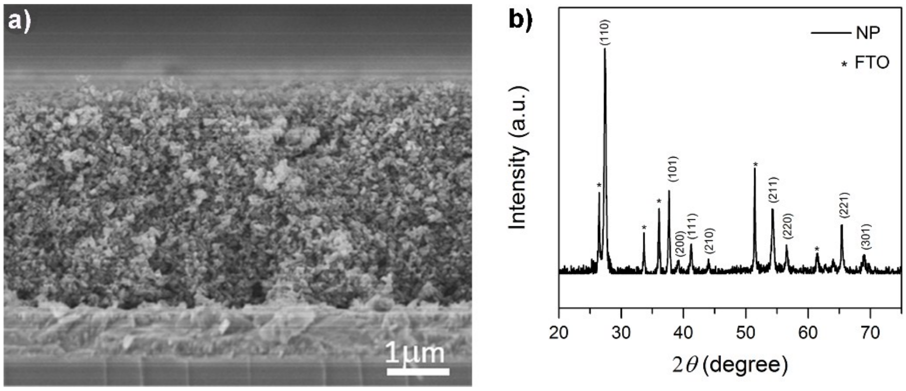

Figure S6. SEM image and XRD pattern of the TiO_2 NP film.

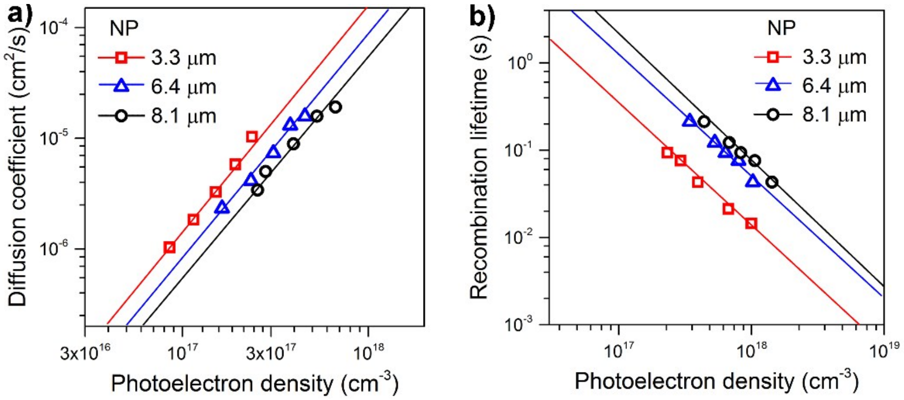

Figure S7. Comparison of electron diffusion coefficients and recombination lifetimes as a function of the photoelectron density for 3.3 μm, 6.4 μm and 8.1 μm thick TiO_2 NP film-based cells.

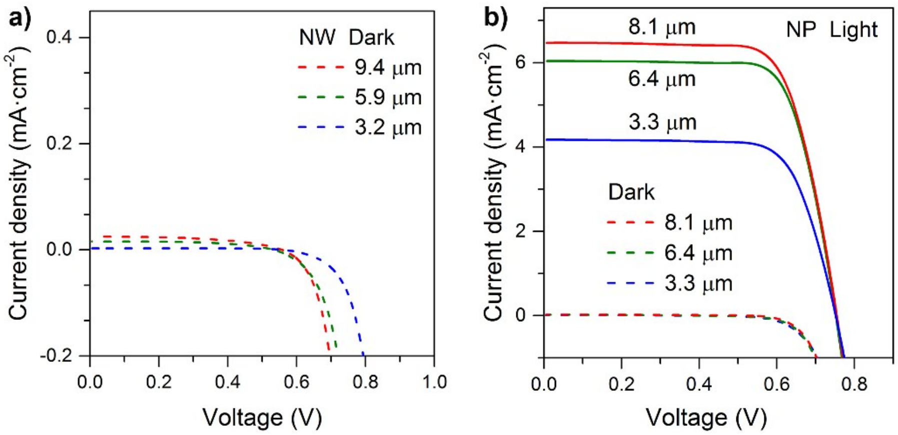

Figure S8. Performance of the TiO_2 NW and NP-based cells. (a) Comparison of the current density–voltage characteristics of the NW-based cells in the dark. (b) Comparison of the current density–voltage characteristics of theTiO_2 NP-based cells under AM 1.5 illumination and in the dark.

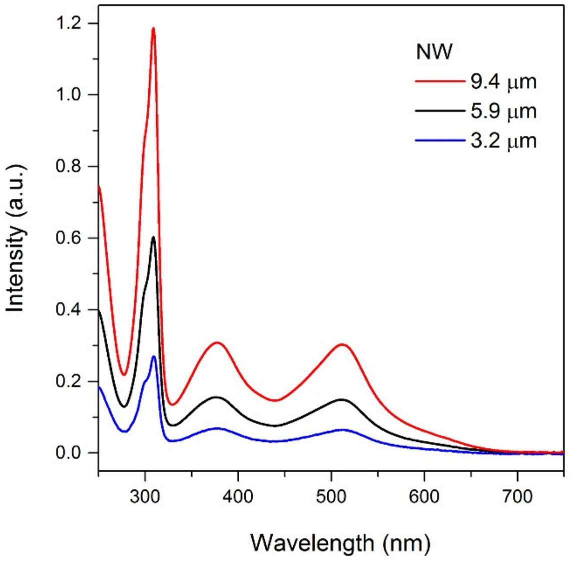

Figure S9. UV-Vis spectra of dye solutions desorbed from the as-prepared TiO_2 NWs for different synthesis times, ranging from 2.5 h to 4.5 h, indicating that the NWs of 4.5 h growth have the highest surface area.

Table S1. Roughness factor of the NWs calculated from UV-Vis spectra of Figure S9; the adsorbed area per dye molecule is assumed to be 1.6 nm^2.

Thickness of film (μm)	Dye adsorption ($nmol/cm^2$)	Roughness factor
3.2	6.46	62
5.9	16.76	161
9.4	23.18	223

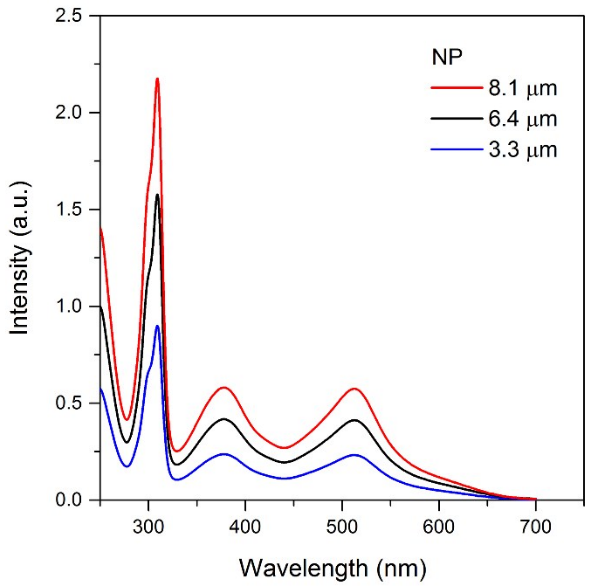

Figure S10. UV-Vis spectra of dye solutions desorbed from the as-prepared TiO_2 NP films of different thicknesses, ranging from 3.3 µm to 8.1 µm.

Table S2. Roughness factor of the NP films calculated from UV-Vis spectra of Figure S10; adsorbed area per dye molecule is assumed to be 1.6 nm^2.

Thickness of film (µm)	Dye adsorption (nmol/cm^2)	Roughness factor
3.3	36.00	347
6.4	62.79	605
8.1	87.99	848

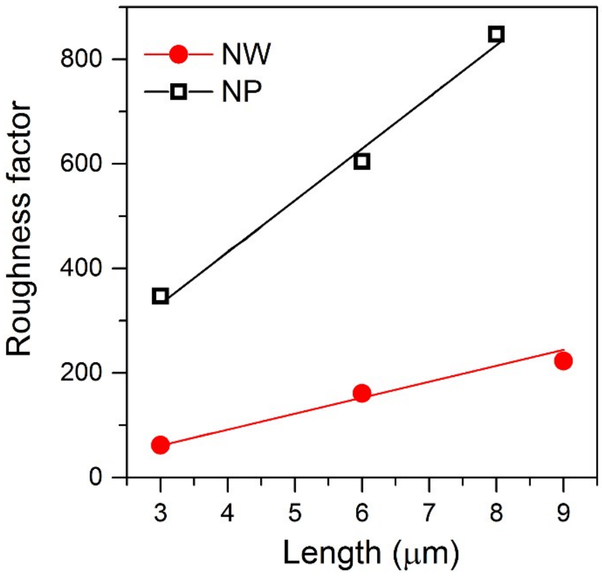

Figure S11. Dependence of the roughness factor on film thickness of TiO_2 NWs and TiO_2 NP films.
